# Heterozygous *THBS2* pathogenic variant causes Ehlers–Danlos syndrome with prominent vascular features in humans and mice

**DOI:** 10.1038/s41431-024-01559-1

**Published:** 2024-03-04

**Authors:** Noam Hadar, Omri Porgador, Idan Cohen, Hilla Levi, Vadim Dolgin, Yuval Yogev, Sufa Sued-Hendrickson, Ilan Shelef, Elena Didkovsky, Marina Eskin-Schwartz, Ohad S. Birk

**Affiliations:** 1 The Morris Kahn Laboratory of Human Genetics at the National Institute of Biotechnology in the Negev and Faculty of Health Sciences, Ben-Gurion University of the Negev, Beer Sheva, Israel; 2 The Shraga Segal Department of Microbiology, Immunology, and Genetics, Faculty of Health Science, Ben-Gurion University of the Negev, Beer Sheva, Israel; 3 Department of Radiology, Soroka Medical Center, and Faculty of Health Sciences, Ben-Gurion University of the Negev, Beer-Sheva, Israel; 4 Department of Pathology, Rabin Medical Center, Petah-Tikva, and Faculty of Medicine, Tel Aviv University, Tel Aviv Israel; 5 Genetics Institute, Soroka University Medical Center, Beer-Sheva, Israel; 6 Faculty of Health Sciences, Ben-Gurion University of the Negev, Beer-Sheva, Israel

**Keywords:** Clinical genetics, Connective tissue diseases, Genetic engineering

## Abstract

Ehlers–Danlos syndromes (EDS) are a group of connective tissue disorders caused by mutations in collagen and collagen-interacting genes. We delineate a novel form of EDS with vascular features through clinical and histopathological phenotyping and genetic studies of a three-generation pedigree, displaying an apparently autosomal dominant phenotype of joint hypermobility and frequent joint dislocations, atrophic scarring, prolonged bleeding time and age-related aortic dilatation and rupture. Coagulation tests as well as platelet counts and function were normal. Reticular dermis displayed highly disorganized collagen fibers and transmission electron microscopy (TEM) revealed abnormally shaped fibroblasts and endothelial cells, with high amount and irregular shape of extracellular matrix (ECM) substance, especially near blood vessels. Genetic analysis unraveled a heterozygous mutation in *THBS2* (NM_003247.5:c.2686T>C, p.Cys896Arg). We generated CRISPR/Cas9 knock-in (KI) mice, bearing the heterozygous human mutation in the mouse ortholog. The KI mice demonstrated phenotypic traits correlating with those observed in the human subjects, as evidenced by morphologic, histologic, and TEM analyses, in conjunction with bleeding time assays. Our findings delineate a novel form of human EDS with classical-like elements combined with vascular features, caused by a heterozygous *THBS2* missense mutation. We further demonstrate a similar phenotype in heterozygous *THBS2*^Cys896Arg^ KI mice, in line with previous studies in *Thbs2* homozygous null-mutant mice. Notably, *THBS2* encodes Thrombospondin-2, a secreted homotrimeric matricellular protein that directly binds the ECM-shaping Matrix Metalloproteinase 2 (MMP2), mediating its clearance. *THBS2* loss-of-function attenuates MMP2 clearance, enhancing MMP2-mediated proteoglycan cleavage, causing ECM abnormalities similar to those seen in the human and mouse disease we describe.

## Introduction

Ehlers–Danlos syndrome (EDS) is a heterogenous group of inherited connective tissue disorders. To date, 14 different clinical types of EDS have been described, featuring dominant or recessive modes of heredity [1–4]. The classic form of EDS comprises joint hypermobility, hyperextensible skin and skin fragility. Aside from the classic EDS phenotype, other subtypes manifest additional attributes, such as vascular or skeletal involvement. Vascular involvement, exemplified by aneurysms and dissections, is a prominent characteristic of vascular type EDS (vEDS), caused by heterozygous pathogenic variants in *COL3A1*. Nonetheless, such vascular involvement has been documented also in other EDS subtypes [1, 5]. The clinical overlap among EDS subtypes highlights the necessity for precise molecular diagnoses in affected individuals.

The molecular pathways leading to EDS are diverse. These include dominantly-inherited structural abnormalities of fibrillar collagens, exerting their effect through haploinsufficiency or dominant negative mechanisms; and recessively-inherited defects in collagen and other extracellular matrix-modifying enzymes [6–8] that instigate disease through loss of protein function.

Through clinical phenotyping, histological analysis and electron microscopy, combined with molecular genetic studies, we describe a dominantly-inherited previously uncharacterized connective tissue disease with features of EDS with prominent vascular involvement, caused by a heterozygous pathogenic variant in *THBS2*.

## Methods

### Patients and clinical evaluation

In accordance with Helsinki principles, participants in this study provided written informed consent as per protocols approved by the Soroka Medical Center institutional review board (approval #5071G) and the Israel National Committee for Human Genetic Studies (approval #920100319). Senior dermatologists and geneticists conducted the phenotyping.

### Genetic analysis

**Fig. 1:**
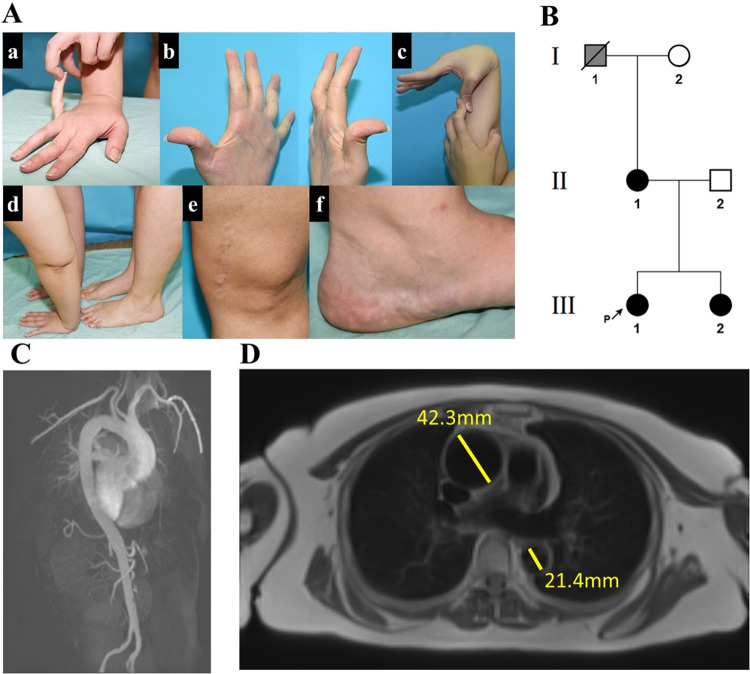
Pedigree and clinical manifestations. **A** Clinical findings (patient III:1) (a–d) joint hypermobility (e) atrophic scarring (f) piezogenic papules. **B** Family tree: black filling denotes affected individuals heterozygous for the *THBS2* NM_003247.5:c.2686T>C p.Cys896Arg variant. Individual I:1 (in gray) passed away at the age of 70 before being clinically or genetically assessed and is likely affected based on the limited available clinical history. **C**, **D** MRA of thorax and abdomen demonstrating dilated aortic arch (patient II:1).

DNA was extracted from peripheral blood leukocytes using E.Z.N.A Blood DNA kit (Omega Bio-tek, Nor-cross, GA, USA). Whole-exome sequencing was performed by Macrogen® using Agilent SureSelect Exome V7 library construction enrichment kit and Illumina NovaSeq6000 system with 150 bp paired-end reads. Raw data reads were aligned to GRCh38 reference genome using BWA-MEM [9] with variant calling facilitated by GATK 4.2.0.0 [10]. Data were analyzed using our in-house software. Our filtration cascade omitted common variants (>0.1%) in multiple databases (gnomAD [11] 2.1.1 and 3.1.2, ALFA [12], 1000 genomes project [13] and our in-house database of 800 exomes and genomes), unless established as pathogenic by ClinVar [14] or the Human Gene Mutation Database (HGMD) [15]. Following filtration, variants shared by both affected individuals (Fig. 1A, III:1 and III:2) were assessed based on previous scientific literature, inspection of shared variants in SRA samples using GeniePool [16], relevant gene expression based on GTEx [17] and Human Protein Atlas [18, 19] and evolutionary conservation based on phyloP [20]. Verification of the pathogenic variant and segregation analysis within the affected kindred was done using Sanger sequencing.

### Histology and electron microscopy

Skin biopsies, obtained from the upper back of patient III:1 and of unrelated age-matched healthy individual and backs and tails of 4 months old mice, were used for histological analysis and for electron microscopy.

Human skin samples and mouse tail and skin samples for histological examination were fixed in 4% paraformaldehyde in 0.1 M phosphate buffer (pH-7.4) overnight at room temperature. Samples were processed for embedding in paraffin and 5 µm-thick sections were stained with hematoxylin-eosin (H&E), or for Masson’s trichrome stain according to manufacturer’s instructions (Sigma-Aldrich). Slides were imaged using an Olympus BX53 upright microscope.

For human samples electron microscopy, skin tissue was fixed with 2.5% glutaraldehyde and 1% paraformaldehyde in Sorenson buffer for 1 h at room temperature (RT), then moved to 4 °C overnight. The tissue was washed with 0.1 M Sodium Cacodylate buffer and stained with 1% Osmium TetraOxide in Cacodylate buffer, and then with 1% uranyl acetate for 1 h at RT. The tissue was then dehydrated and embedded in Epon 812. Ultra-thin (75 nm) sections were cut using Leica UC7 ultramicrotome. Sections were transferred to copper grids and visualized using a Talos L120C Transmission Electron Microscope at an accelerating voltage of 120 kV. Mice samples were visualized using Talos F200C transmission electron microscope operating at 200 kV.

### Generation of Thbs2 CRISPR/Cas9-mediated mutant mice

Knock-in C57BL/6J mice, harboring p.Cys896Arg mutation in the mouse *Thbs2* ortholog, were generated at The National Genetically Engineered Mouse Models (GEMM) Unit at the Hebrew University of Jerusalem using the CRISPR/Cas9 system [21, 22]. We designed sequences of sgRNA (5’-AUUGUCAUCAUCAGAGUCGC-3’) and ssODN (5’-TGCCCATACATCTCCAACTCCAACCAGGCTGACCATGACAACGACGGCAAGGGCGATGCACGCGACTCTGATGATGACAATGATGGTGTTCCAGATGACAGGGACAACTGTCGGCTTGTG- 3’) utilizing the Benchling platform (https://www.benchling.com/crispr) and procured these from IDT (Integrated DNA Technologies, Coralville, Iowa, USA). Sanger-sequencing-confirmed F0 male knock-in heterozygote was crossbred with wild type (WT) C57BL/6J mice to generate F1 mice, which were bred with WT mice to generate F2 mice, resulting in littermate cohorts of WT and heterozygotes for subsequent experimentation.

### Mice maintenance and ethics statement

Mice were maintained in the animal facility of the Ben-Gurion University of the Negev on a 12:12 h light/dark schedule at temperatures of 20–24 °C and 30–70% humidity with no food limitation. In-vivo experiments were preformed according to the National Institutes of Health guidelines for care and use of laboratory animals (NIH publications No. 8023, revised 1978) and according to the guidelines of the Israeli Council on Animal Care. Experimental protocols were approved by the Committee on Animal Care and Use of Ben-Gurion University of the Negev.

### Bleeding time assay

Standard 50 ml tubes were modified generating 5 air vents and one central hole in the tube lid. Standard food pellets were added to the tube as a distraction for the mice. Tubes were positioned horizontally on a receptacle, with a second 50 ml tube containing PBS (Dulbecco’s Phosphate Buffered Saline without magnesium or calcium, Sartorius™) vertically bellow its lid. Each mouse was individually placed into the modified tube, aligning its tail with the hole in the lid, so that the tail was leveled with the mouse’s heart. Then, under video surveillance, a 0.5 cm portion of the mouse tail tip was severed and immersed in the PBS tube. Bleeding duration was assessed as the time from the tail tip was cut to the visual cessation of blood flow in the tube, or to a maximum of 12 min, after which manual pressure was applied to the tail to arrest bleeding. The assay was conducted on eight 4-month old F2 male mice, born on the same day and derived from two litters, each consisting of 2 heterozygotes and 2 WT mice.

## Results

### Clinical findings

The index case (patient III:1, Fig. 1A), a female of Ashkenazi Jewish ancestry in her late twenties, presented with a history of multiple joint dislocations (including jaw, shoulder, thigh, knee and ankle joints) and tendon rupture (anterior talo-fibular ligament; ATFL), easy bruising, prolonged wound healing, and lower limb muscle fatigue following exercise. Her physical examination revealed a BMI of 35 and prominent joint hypermobility, with Beighton score of 5/9: passive dorsiflexion and hyperextension of the fifth MCP joint beyond 90° (2/2 points); passive apposition of the thumb to the flexor aspect of the forearm (2/2 points); active forward flexion of the trunk with the knees fully extended so that the palms of the hands rest flat on the floor (1/1 point). Also evident were atrophic scarring and the presence of piezogenic papules on the medial aspect of her feet (Fig. 1A). Significant skin hyperextensibility was not observed. The sister (III:2, in her thirties) and mother (II:1, in her fifties) of III:1 had similar findings and her maternal grandfather (I:1) had cerebral aneurysms and died of dissection of the abdominal aorta during the seventh decade of life (Fig. 1B). Notably, III:2 suffered from symphysiolysis while giving birth and reported increased flexibility while breastfeeding. Her child was not evaluated due to parents’ preference.

Significantly, each of the three affected patients has had at least five imaging tests in the last ten years, including CTs, X-rays, MRIs and skeletal scintigraphy for each. Patient III:1 was diagnosed with bilateral greater saphenous vein insufficiency (GSVI). II:1 was diagnosed with unilateral GSVI. Cardiac echocardiogram of II:1 demonstrated a thickened mitral valve and a dilated aortic arch, which was further displayed by chest MRI angiography to be 4.2 cm wide (Fig. 1C, D). Thoracic aorta diameter was normal. Brain MRI angiography done for III:1 in her twenties and for II:1 in her fifties were normal. There was no evidence of cardiomyopathy in any of the patients.

Prolonged bleeding was evident in the affected individuals: II:1 reported she had bled extensively while delivering both of her daughters. Furthermore, following skin punch biopsy from III:1 and a healthy matched control, III:1 experienced continuous bleeding for over 20 min while the control did not bleed after the procedure. Platelet counts, prothrombin time, and INR were within normal limits in all available affected individuals. Additional tests done for III:1 and III:2, including Von Willebrand factor, clotting factor VIII, Fibrinogen, and platelet function using the INNOVANCE® PFA-200 System, did not indicate any clotting abnormalities (Supplementary Table 1).

### Molecular genetics

**Fig. 2:**
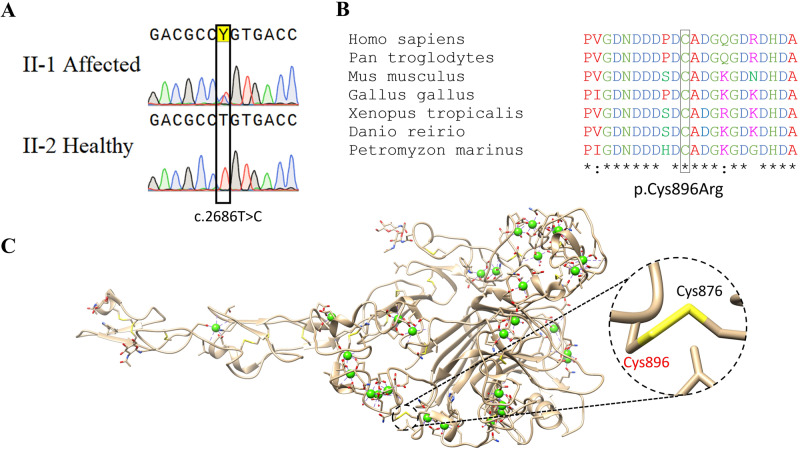
The *THBS2* pathogenic variant. **A** Sanger sequencing of affected (II-1) and healthy (II-2) individuals. The *THBS2* NM_003247.5:c.2686T>C variant is marked by a black rectangle. **B** Conservation analysis of the locus surrounding the *THBS2* p.Cys896Arg variant. Symbols below indicate conservation: asterisk for complete identity; colon for high similarity and blank for none. (Clustal-Omega - https://www.ebi.ac.uk/Tools/msa/clustalo) **C** 3D model of the crystalized THBS2 protein showing the Cys876-Cys896 disulfide bond eliminated by the THBS2 p.Cys896Arg variant (marked in red) (https://www.rcsb.org/structure/1YO8).

Testing patient III:1, no variants were identified within the coding sequence of a panel of 15 known EDS-related genes (*ADAMTS2, ATP7A, CHST14, COL12A1, COL1A1, COL1A2, COL3A1, COL5A1, COL5A2, CRTAP, FKBP14, FLNA, P3H1, PLOD1, SLC39A13*). Analysis of whole-exome sequencing data from III:1 and III:2 did not identify pathogenic variants in genes previously associated with EDS (including *AEBP1*, not tested in the above panel), and revealed 43 heterozygous variants shared by the two affected individuals, that were not ruled out by our pipeline. However, further bioinformatics analysis showed that most of the 43 variants were frequent in Ashkenazi Jews, a population highly represented in our in-house and public databases, and were therefore dismissed. All other variants were ruled out through segregation analysis in the extended family, except for *THBS2* NM_003247.5:c.2686T>C p.Cys896Arg, that stood out as a plausible disease-causing pathogenic variant: it was not found in over 260,000 controls from multiple databases, including gnomAD, in which >7000 samples are of Ashkenazi Jews. Furthermore, Sanger sequencing demonstrated that the *THBS2* variant segregated within the studied family consistent with fully-penetrant dominant inheritance (Fig. 2A); although a DNA sample and detailed phenotyping were not available for the deceased individual I:1, I:2 was shown not harbor the pathogenic variant, suggesting that the *THBS2* variant was likely inherited from I:1, who died of aortic dissection.

The p.Cys896Arg variant lies within a highly evolutionary-conserved region of *THBS2* (Fig. 2B). According to the solved protein structure, it putatively eliminates a critical disulfide bond formed by the 876^th^ and the 896^th^ amino acids of THBS2 (Fig. 2C). Notably, *THBS2* is highly expressed in fibroblasts and arteries, and has been clearly linked to extra cellular matrix (ECM) remodeling. Moreover, *Thbs2* null mutant mice have been previously shown to display an EDS-like phenotype with prolonged bleeding time [23].

### Heterozygous *Thbs2* p.Cys896Arg CRISPR/Cas9 knock-in mice

**Fig. 3:**
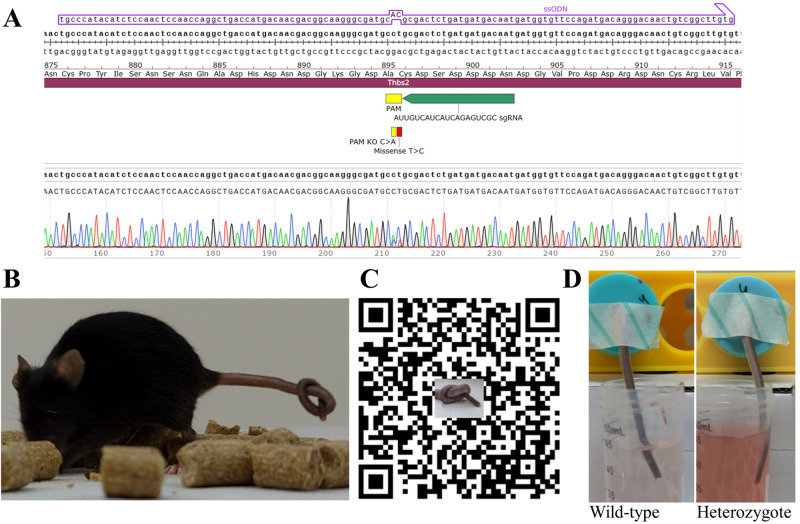
CRISPR/Cas9 *THBS2* p.Cys896Arg heterozygous knock-in mice. **A** Schematic representation of the CRISPR/Cas9 modification used to generate knock-in mice with the c.2686T>C variant and Sanger sequencing result from F0 knock-in mice. **B** An image and a video (**C**) of a F0 knock-in female mouse having a knot in her tail while keeping calm. Notably, wild type mice cannot have their tails tied in that manner. **D** Bleeding time assay for wild-type (left) and heterozygote (right) male littermates at the time of experiment termination. All wild-type mice stopped bleeding after less than 4 min while mutant mice bled over 11 min.

Heterozygote knock-in *Thbs2* p.Cys896Arg mice that we generated (Fig. 3A) initially appeared healthy and normal. However, by the age of 3 months they were easily differentiated from WT mice simply by tactile interaction such as grabbing their tail: the tails of heterozygous mice were remarkably flexible, so that they could be effortlessly knotted at the ends without causing any visible distress to the mice (Fig. 3B, C). Moreover, when lifting heterozygous mice by the tail and subsequently releasing it, the tail preserved its arched form, nearly mirroring the shape it assumed while being held (Fig. 3C). At the age of 4 months, the mice were significantly more flexible and felt different when being handled. Notably, pregnancies enhanced the disease process: female mutant mice became flexible following parturition, exhibiting hyper-flexibility (both per subjective assessment and by ability to tie a knot at tail ends) as early as 2 months of age, to an extent similar to that evident in male and non-pregnant female heterozygous mice at age 4 months.

A stark contrast was observed in the bleeding times of the WT and mutant mice. The four WT mice tested exhibited bleeding times of under 4 min, specifically ranging from 200 to 240 s. Conversely, one mutant mouse had bled for 11 min, and the other three for periods exceeding 12 min, which necessitated the termination of the experiment for these subjects (Fig. 3D).

### Light microscopy

**Fig. 4:**
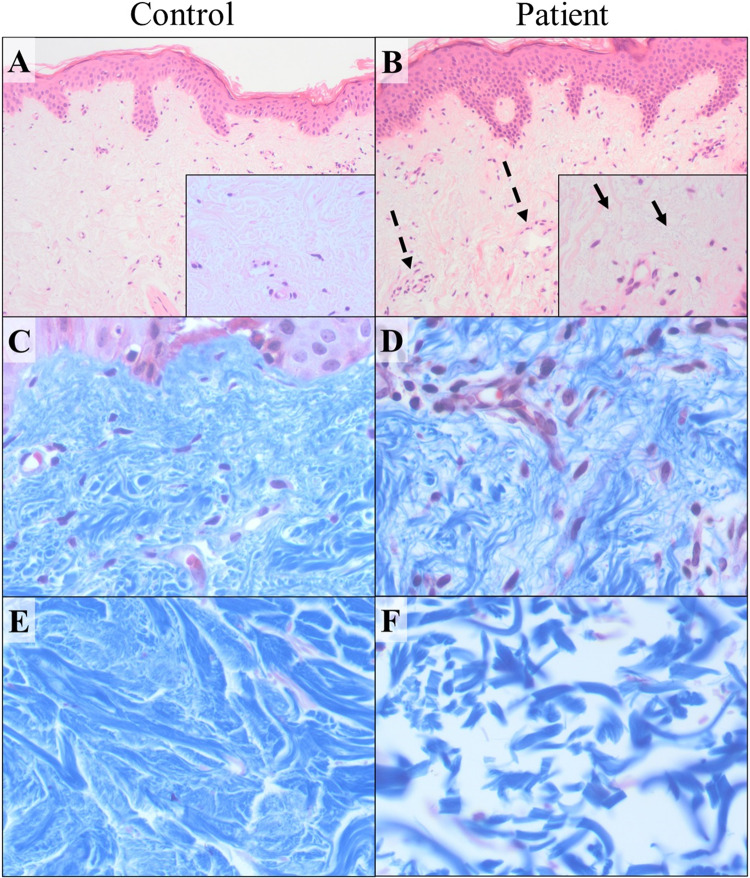
Histology of upper back skin punch biopsies from patient III:1 and an age-matched control. H&E staining (**A** control, **B** patient; X100, insert X400) demonstrate multiple dilated blood vessels with mild perivascular lymphocytic infiltrate in the reticular dermis (dotted arrows) with mucinous material between disorganized collagen fibers (full arrows). Masson’s trichrome staining of the same subjects highlighting the disorganized and thin collagen fibers in the papillary dermis (**C** – control, **D** – patient; X400) and, most prominently, in the reticular dermis (**E** – control, **F** – patient; X400).

Human studies - histological examination of skin biopsy of III:1: hematoxylin and eosin (H&E) staining revealed thin and disorganized collagen fibers in the reticular dermis, with mucinous material observed in the interspaces. Additionally, the reticular dermis exhibited an increased presence of dilated blood vessels in contrast to the control sample (Fig. 4A, B). Masson trichrome (MT) staining highlighted the disorganized collagen fibers in the papillary dermis (Fig. 4C, D) and even more so in the reticular dermis (Fig. 4E, F). Notably, findings in the reticular dermis demonstrate that while both samples were prepared using the same protocol, collagen fibers in the mutant sample were highly fragmented. Periodic Acid-Schiff (PAS) staining was negative. Alcian blue staining demonstrated patchy positivity, suggesting the presence of mucin within the substance observed between the collagen fibers (data not shown). Examination of the epidermis did not yield any significant findings.

Mouse histological examination was performed for both male and female, WT and heterozygous mice using H&E and MT staining (Supplementary Fig. 1). H&E (Supplementary Fig. 1A–D) and MT findings were in line with those of the human subjects, demonstrating disorganized collagen fibers in the reticular dermis in both male and female mutant mice.

### Electron microscopy

TEM studies of human affected individual III:1 revealed abnormally-shaped fibroblasts and endothelial cells, as well as unusually high quantity and irregular shape of extracellular matrix (ECM) substance, particularly in the proximity of blood vessels (Supplementary Fig. 2A, E). When comparing human control and mutant collagen fibrils, no unusual differences were found (Supplementary Fig. 2B, F).

However, a stark contrast was observed when assessing the mutant mice collagen fibrils, which were larger and displayed irregular contours, evidently in the tail samples (Supplementary Fig. 2C, G), and even much more so in skin samples (Supplementary Fig. 2D, H). The mice skin samples were devoid of blood vessels.

## Discussion

We describe a three generation family with multiple subjects affected by a dominantly inherited connective tissue disorder, that can be clinically classified as EDS: according to criteria of the 2017 International Classification of EDS [1], the disease we delineate is reminiscent of classic EDS phenotype per Beighton score [24] (1 major criterion - 5/9, and 4 minor criteria – easy bruising, soft doughy skin, multiple sprains and subluxations and positive family history of a similar disorder), albeit with prominent vascular features.

The extracellular matrix (ECM) comprises structural proteins, such as collagens, fibronectin and laminin, among multiple other protein constituents. These elements are paramount for maintaining the ECM’s integrity and organization [25]. Of these, matricellular proteins are the non-structural ECM components, influencing ECM assembly through cell-matrix interactions and cellular signaling [26]. To date, pathogenic variants in multiple genes encoding fibrillar collagens, collagen and other extracellular matrix-modifying enzymes [6–8] as well as extracellular matrix proteins [1, 27], have been found to cause connective tissue disorders that can be defined as subtypes of EDS. Through our complementary human and mouse studies, we demonstrate that the novel form of EDS we describe is caused by a heterozygous missense pathogenic variant in *THBS2*. Thrombospondin-2, encoded by *THBS2* (MIM 188061), is a secreted homotrimeric matricellular protein [28–31]. It was shown to directly bind Matrix Metalloproteinase 2 [32] (*MMP2*, MIM 120360), an ECM -shaping metalloproteinase and angiogenesis modifier [33], and to mediate its clearance [34]. Moreover, it has been shown in-vitro and in mice that THBS2 loss-of-function modulates MMP2 clearance, leading to higher amounts of ECM MMP2, known to enhance MMP-2-mediated cleavage of the proteoglycan decorin [35, 36]causing extracellular matrix abnormalities [31, 34, 37] similar to those seen in the histological and EM studies of the human disease we describe. As decorin has been shown to interact with collagen 12 [38], it is thus also feasible that the disease process involves downstream effects on collagen 12. Further studies, beyond the scope of this manuscript, are in place to delineate the detailed downstream disease mechanism.

Of note, a recent manuscript suggests that hypermobility presentation may be dependent on folate status affecting MMP2 molecular pathways, and that EDS-related hypermobility could possibly be modulated by 5-methyltetrahydrofolate supplementation [36]. Thus, future studies are in place to assess possible effects of 5-methyltetrahydrofolate supplementation in both the human and mouse *THBS2* disease we describe.

Findings derived from GTEx [17] regarding THBS2 show that the protein is mainly expressed in large blood vessels such as the aorta, coronary arteries and tibial artery. Patient I:1 died from abdominal aorta dissection at the age of 70 and II:1 displayed enlarged ascending aortic arch at 50 years of age (Fig. 1C, D). Progressive aortic root dilatation, spontaneous rupture of large arteries and intracranial aneurysms have only rarely been reported in classical EDS patients [39–41], and increased tissue vascularity is not a feature of this disorder, suggesting that EDS caused by the heterozygous *THBS2* pathogenic variant represents a distinct clinical entity.

Homozygous null-mutant *Thbs2* mice display a variety of connective tissue abnormalities, including lax skin, ligaments and tendons, disorganized abnormally shaped collagen fibers and increased number of small to medium sized blood vessels in the skin and subcutaneous tissue, as well as a bleeding diathesis and enhanced cardiac aging resulting in dilated cardiomyopathy, thereby highlighting the important role of this protein in connective tissue homeostasis [23, 28]. Many features observed in the affected heterozygote human subjects recapitulate the findings in the mouse *Thbs2* knockout (KO) model. However, the fact that the affected humans were heterozygous for the *THBS2* mutation made the THBS2 variant into a non-obvious candidate for causing the described disease. Thus, we resorted to generate CRISPR/Cas9-KI *Thbs2* p.Cys896Arg heterozygous mice, recapitulating the previously described findings in homozygous KO *Thbs2* mice in each of our assays.

Both the previously reported *Thbs2* homozygous KO mice and the heterozygous *Thbs2* p.Cys896Arg KI mutant mice we generated displayed disordered collagen fibers with larger diameter and abnormal contour that was different in scale when comparing skin and tail tissues (Supplementary Fig. 2) [23]. However, the collagen fibers in the human samples affected by the disease did not show significant differences compared to those in the healthy control; while cauliflower-like collagen fibrils are clearly evident in the skin samples of mutant mice, they are less pronounced in their tail samples and absent in the human mutant samples. These differences suggest that additional factors may influence the extent to which THBS2 affects collagen modeling, potentially including variations in collagen type composition between humans and mice and moreover in their different tissues. Understanding the cause of these differences may thus be helpful in discovering ways to mitigate the disease.

Interestingly, echocardiography of the affected subjects did not reveal the presence of age-related cardiomyopathy, observed in the KO mouse model, possibly reflecting the relatively young age of the examined family members, as well as possible partial penetrance of this feature in this disorder. Nevertheless, at this point it seems reasonable to include long-term cardiac assessment in the clinical follow-up of patients with *THBS2*-related EDS as well as further cardiac evaluation of the KI mice.

Most notably, our findings demonstrate that the *THBS2* p.Cys896Arg variant, being sufficient to cause a disease in a heterozygous state, has a dominant effect. More specifically, THBS2 is a homotrimer and the mutated cysteine at the 896^th^ position of the protein is known to create a disulfide bond at the 876^th^ position (Fig. 2). This missense mutation putatively alters the protein’s structure, yet still enables it to bind other functioning proteins and disable them. Capability of *THBS2* mutations to have a dominant effect is well in line with the fact that somatic heterozygous *THBS2* pathogenic variants can serve as cancerous driver, enabling tumor expansion through the disorganized extracellular matrix caused by the same processes shown in this study [42–44]. It is plausible that cancer cells producing malformed *THBS2* monomers can cause local organ dissemination and increase invasiveness. Moreover, it may be of interest to test whether germline *THBS2* pathogenic variants might facilitate metastatic processes in tumors that are located in collagen-enriched organs. Notably, no malignancies were reported in the affected individuals of all generations.

### Supplementary information

Supplementary Figure 1. Histology of back skin from THBS2 knock-in mice.

Supplementary figure 2. Transmission electron microscopy of human and mouse THBS2 p.Cys896Arg heterozygotes and wild-type controls.

Platelet function analysis

## Data Availability

The datasets generated and/or analyzed during the current study are available from the corresponding author on reasonable request.

## References

[1] Malfait F, Francomano C, Byers P, Belmont J, Berglund B, Black J (2017). The 2017 international classification of the Ehlers–Danlos syndromes. Am J Med Genet C Semin Med Genet.

[2] Hebebrand M, Vasileiou G, Krumbiegel M, Kraus C, Uebe S, Ekici AB (2019). A biallelic truncating AEBP1 variant causes connective tissue disorder in two siblings. Am J Med Genet A.

[3] Blackburn PR, Xu Z, Tumelty KE, Zhao RW, Monis WJ, Harris KG (2018). Bi-allelic Alterations in AEBP1 Lead to Defective Collagen Assembly and Connective Tissue Structure Resulting in a Variant of Ehlers-Danlos Syndrome. Am J Hum Genet.

[4] Alazami AM, Al-Qattan SM, Faqeih E, Alhashem A, Alshammari M, Alzahrani F (2016). Expanding the clinical and genetic heterogeneity of hereditary disorders of connective tissue. Hum Genet.

[5] Brady AF, Demirdas S, Fournel-Gigleux S, Ghali N, Giunta C, Kapferer-Seebacher I (2017). The Ehlers-Danlos syndromes, rare types. Am J Med Genet C Semin Med Genet.

[6] Hyland J, Ala-Kokko L, Royce P, Steinmann B, Kivirikko KI, Myllylä R (1992). A homozygous stop codon in the lysyl hydroxylase gene in two siblings with Ehlers–Danlos syndrome type VI. Nat Genet.

[7] Malfait F, Syx D, Vlummens P, Symoens S, Nampoothiri S, Hermanns-Lê T (2010). Musculocontractural Ehlers-Danlos Syndrome (former EDS type VIB) and adducted thumb clubfoot syndrome (ATCS) represent a single clinical entity caused by mutations in the dermatan-4-sulfotransferase 1 encoding CHST14 gene. Hum Mutat.

[8] Mao JR, Taylor G, Dean WB, Wagner DR, Afzal V, Lotz JC (2002). Tenascin-X deficiency mimics Ehlers-Danlos syndrome in mice through alteration of collagen deposition. Nat Genet.

[9] Li H, Durbin R (2009). Fast and accurate short read alignment with Burrows-Wheeler transform. Bioinformatics..

[10] Poplin R, Ruano-Rubio V, DePristo MA, Fennell TJ, Carneiro MO, van der Auwera GA, et al. Scaling accurate genetic variant discovery to tens of thousands of samples. bioRxiv. 2017;201178. Available from: 10.1101/201178.

[11] Karczewski KJ, Francioli LC, Tiao G, Cummings BB, Alföldi J, Wang Q (2020). The mutational constraint spectrum quantified from variation in 141,456 humans. Nature..

[12] Phan L, Jin Y, Zhang H, Qiang W, Shekhtman E, Shao D, et al. Allele Frequency Aggregator. Available from: https://www.ncbi.nlm.nih.gov/snp/docs/gsr/alfa/#citing-this-project.

[13] Auton A, Abecasis GR, Altshuler DM, Durbin RM, Bentley DR, Chakravarti A, et al. A global reference for human genetic variation. Nature. 2015;526:68–74. 10.1038/nature15393 PMC4750478 26432245

[14] Landrum MJ, Lee JM, Benson M, Brown GR, Chao C, Chitipiralla S (2018). ClinVar: Improving access to variant interpretations and supporting evidence. Nucleic Acids Res.

[15] Stenson PD, Mort M, Ball EV, Shaw K, Phillips AD, Cooper DN (2014). The Human Gene Mutation Database: Building a comprehensive mutation repository for clinical and molecular genetics, diagnostic testing and personalized genomic medicine. Hum Genet Hum Genet.

[16] Hadar N, Weintraub G, Gudes E, Dolev S, Birk OS (2023). GeniePool: genomic database with corresponding annotated samples based on a cloud data lake architecture. Database.

[17] Lonsdale J, Thomas J, Salvatore M, Phillips R, Lo E, Shad S (2013). The Genotype-Tissue Expression (GTEx) project. Nat Genet.

[18] Uhlen M, Fagerberg L, Hallstrom BM, Lindskog C, Oksvold P, Mardinoglu A (2015). Tissue-based map of the human proteome. Science.

[19] Thul PJ, Akesson L, Wiking M, Mahdessian D, Geladaki A, Ait Blal H (2017). A subcellular map of the human proteome. Science.

[20] Pollard KS, Hubisz MJ, Rosenbloom KR, Siepel A (2010). Detection of nonneutral substitution rates on mammalian phylogenies. Genome Res.

[21] Ran FA, Hsu PD, Wright J, Agarwala V, Scott DA, Zhang F (2013). Genome engineering using the CRISPR-Cas9 system. Nat Protoc.

[22] Alghadban S, Bouchareb A, Hinch R, Hernandez-Pliego P, Biggs D, Preece C (2020). Electroporation and genetic supply of Cas9 increase the generation efficiency of CRISPR/Cas9 knock-in alleles in C57BL/6J mouse zygotes. Sci Rep.

[23] Kyriakides TR, Zhu YH, Smith LT, Bain SD, Yang Z, Lin MT (1998). Mice that lack thrombospondin 2 display connective tissue abnormalities that are associated with disordered collagen fibrillogenesis, an increased vascular density, and a bleeding diathesis. J Cell Biol.

[24] Juul-Kristensen B, Røgind H, Jensen DV, Remvig L (2007). Inter-examiner reproducibility of tests and criteria for generalized joint hypermobility and benign joint hypermobility syndrome. Rheumatology..

[25] Frantz C, Stewart KM, Weaver VM (2010). The extracellular matrix at a glance. J Cell Sci..

[26] Bornstein P, Sage EH. Matricellular proteins: Extracellular modulators of cell function. Curr Opin Cell Biol. 2002;14:608–16. 10.1016/s0955-0674(02)00361-7 12231357

[27] Chiarelli N, Ritelli M, Zoppi N, Colombi M (2019). Cellular and molecular mechanisms in the pathogenesis of classical, vascular, and hypermobile ehlers‒danlos syndromes. Genes.

[28] Bornstein P, Agah A, Kyriakides TR The role of thrombospondins 1 and 2 in the regulation of cell-matrix interactions, collagen fibril formation, and the response to injury. 36, International Journal of Biochemistry and Cell Biology. Elsevier Ltd; 2004. p. 1115–25. 10.1016/j.biocel.2004.01.012 15094126

[29] Kyriakides TR, Zhu YH, Yang Z, Bornstein P (1998). The distribution of the matricellular protein thrombospondin 2 in tissues of embryonic and adult mice. J Histochem Cytochem.

[30] Bornstein P, Armstrong LC, Hankenson KD, Kyriakides TR, Yang Z. Thrombospondin 2, a matricellular protein with diverse functions. Matrix Biol. 2000;19:557–68. 10.1016/s0945-053x(00)00104-9 11102746

[31] Bornstein P, Kyriakides TR, Yang Z, Armstrong LC, Birk DE (2000). Thrombospondin 2 modulates collagen fibrillogenesis and angiogenesis. J Investig Dermatol Symp Proc.

[32] Yang Z, Kyriakides TR, Bornstein P (2000). Matricellular proteins as modulators of cell-matrix interactions: Adhesive defect in thrombospondin 2-null fibroblasts is a consequence of increased levels of matrix metalloproteinase-2. Mol Biol Cell.

[33] Henriet P, Emonard H. Matrix metalloproteinase-2: Not (just) a “hero” of the past. Biochimie. 2019;166:223–32. 10.1016/j.biochi.2019.07.019 31362036

[34] Yang Z, Strickland DK, Bornstein P (2001). Extracellular Matrix Metalloproteinase 2 Levels are Regulated by the Low Density Lipoprotein-related Scavenger Receptor and Thrombospondin 2. J Biol Chem.

[35] Imai K, Hiramatsu A, Fukushima D, Pierschbacher MD, Okada Y (1997). Degradation of decorin by matrix metalloproteinases: identification of the cleavage sites, kinetic analyses and transforming growth factor-beta1 release. Biochem J.

[36] Courseault J, Kingry C, Morrison V, Edstrom C, Morrell K, Jaubert L (2023). Folate-dependent hypermobility syndrome: A proposed mechanism and diagnosis. Heliyon..

[37] Bornstein P (2001). Thrombospondins as matricellular modulators of cell function. J Clin Investig Am Soc Clin Investig.

[38] Font B, Eichenberger D, Rosenberg LM, Van Der Rest M (1996). Characterization of the interactions of type XII collagen with two small proteoglycans from fetal bovine tendon, decorin and fibromodulin. Matrix Biol..

[39] Wenstrup RJ, Meyer RA, Lyle JS, Hoechstetter L, Rose PS, Levy HP (2002). Prevalence of aortic root dilation in the Ehlers-Danlos syndrome. Genet Med.

[40] McDonnell NB, Gorman BL, Mandel KW, Schurman SH, Assanah-Carroll A, Mayer SA (2006). Echocardiographic findings in classical and hypermobile Ehlers-Danlos syndromes. Am J Med Genet.

[41] Atzinger CL, Meyer RA, Khoury PR, Gao Z, Tinkle BT (2011). Cross-sectional and longitudinal assessment of aortic root dilation and valvular anomalies in hypermobile and classic Ehlers-Danlos syndrome. J Pediatrics.

[42] Yang Z, Wu H, Zhang K, Rao S, Qi S, Liu M (2022). Circ_0007580 knockdown strengthens the radiosensitivity of non-small cell lung cancer via the miR-598-dependent regulation of THBS2. Thorac Cancer.

[43] Wang L, Feng L, Liu L, Han J, Zhang X, Li D (2023). Joint effect of THBS2 and VCAN accelerating the poor prognosis of gastric cancer. Aging.

[44] Bao Y, Yan E, Wang N. Evaluation of GREM1 and THBS2 as prognostic markers in in non-small cell lung cancer. J Cancer Res Clin Oncol. 2023;149:7849–56. 10.1007/s00432-023-04746-7 PMC11796594 37037928

